# Stay at Home and Teach: A Comparative Study of Psychosocial Risks Between Spain and Mexico During the Pandemic

**DOI:** 10.3389/fpsyg.2020.566900

**Published:** 2020-09-30

**Authors:** Vicente Prado-Gascó, María T. Gómez-Domínguez, Ana Soto-Rubio, Luis Díaz-Rodríguez, Diego Navarro-Mateu

**Affiliations:** ^1^Department of Social Psychology, Faculty of Psychology, University of Valencia, Valencia, Spain; ^2^Department of Inclusive Education, Socio-Community Development and Occupation Sciences, Faculty of Education Sciences, Catholic University of Valencia, Valencia, Spain; ^3^Department of Personality, Assessment and Psychological Treatments, Faculty of Psychology, University of Valencia, Valencia, Spain; ^4^Department of Postgrad of Unity of Culiacán, Pedagogical University of the State of Sinaloa, Sinaloa, Mexico

**Keywords:** psychosocial risks, teachers, coronavirus disease 2019 (COVID-19), Spain, Mexico, pandemic (COVID-19)

## Abstract

**Context:**

The emergency situation caused by coronavirus disease 2019 (COVID-19) has affected different facets of society. Although much of the attention is focused on the health sector, other sectors such as education have also experienced profound transformations and impacts. This sector is usually highly affected by psychosocial risks, and this could be aggravated during the current health emergency. Psychosocial risks may cause health problems, lack of motivation, and a decrease of effectiveness at work, which in turn affect the quality of teaching. Despite their importance, there are hardly any studies that analyze psychosocial risks of non-university teachers during a health emergency such as that caused by COVID-19.

**Objectives:**

The aim of this study was to analyze the perception of COVID-19 and the psychosocial risks of non-university teachers comparing Spain and Mexico during the state of alarm caused by COVID-19.

**Methods:**

Data were collected from 421 non-university teachers (80.2% women; 56.3% from Mexico, 43.7% from Spain) aged 24–60 (*M* = 39.32, *SD* = 10.21) *via* a self-completed questionnaire during the pandemic from March to April 2020.

**Results:**

Data analysis suggests that inequity is the most important risk, followed by work overload. Teachers appear to be moderately satisfied with the information on COVID-19 and the measures taken, while their satisfaction with the available resources is lower. When comparing the two countries, significant differences can be observed in every risk considered except for social support, with lower levels in Mexican teachers compared to Spanish ones. In the case of the perception of COVID-19 and its impact, the perception in general of levels of information, measures, and resources is better among Mexican teachers than among Spanish ones, who present higher scores of the impact of the health emergency.

**Conclusion:**

The results underline the importance of the professional’s perception of resources during a health emergency, which could prevent to some extent burnout and possible alterations associated with it. The measures taken by the responsible entities and the provision of information do affect teachers not only directly but also indirectly by making them more vulnerable to psychosocial risks that could affect their health and professional performance, thus affecting students as well.

## Introduction

### Coronavirus Disease 2019 Pandemic

On January 09, 2020, the China Center for Disease Control and Prevention (China CDC) reported that a novel coronavirus had been detected as the causative agent for 15 of the 59 cases of pneumonia (European Centre for Disease Prevention and Control, 2020c; Holmes, 2020). On January 30, 2020, the World Health Organization (WHO) declared this first outbreak of novel coronavirus a “public health emergency of international concern” (World Health Organization, 2020a). On March 11, 2020, the Director General of the WHO declared coronavirus disease 2019 (COVID-19) a global pandemic (European Centre for Disease Prevention and Control, 2020d; World Health Organization, 2020b). As of March 25, 2020, all European Union (EU)/European Economic Area (EEA) countries and more than 150 countries worldwide are affected (World Health Organization, 2020a,World Health Organization, 2020b). As of April 15, 2020, many EU/EEA countries started to adjust their response measures (i.e., gradual opening of school, small shops, and other businesses, etc.) (European Centre for Disease Prevention and Control, 2020a).

Data from the EU/EEA show that around 20–30% of diagnosed COVID-19 cases are hospitalized, and 4% have severe illness. Hospitalization rates are higher for those aged 60 years and above and for those with other underlying health conditions (European Centre for Disease Prevention and Control, 2020b).

Since December 31, 2019, and as of May 23, 2020, 5,175,476 cases of COVID-19 have been reported, including 338,039 deaths (European Centre for Disease Prevention and Control, 2020b). As this is a new virus, no vaccine is currently available; it may be many months or even more than a year before a vaccine has been tested and is ready for use in humans (European Centre for Disease Prevention and Control, 2020d).

Main global data on cases and death are shown in Table 1.

**TABLE 1:** Data on main countries affected by COVID-19 around the world.

Continent	Total cases	Total deaths	Countries with more cases	Cases by country	Countries with more deaths	Deaths per country
Africa	103,801	3,183	South Africa	20,125	Egypt	707
			Egypt	15,786	Algeria	582
			Algeria	7,918	South Africa	397
			Morocco	7,332	Nigeria	221
			Nigeria	7,261	Morocco	197
Asia	903,105	26,567	Turkey	154,500	Iran	7,300
			Iran	131,652	China	4,638
			India	125,101	Turkey	4,276
			China	84,081	India	3,720
			Saudi Arabia	67,719	Indonesia	1,326
America	2,365,427	139,960	United States	1,601,434	United States	96,007
			Brazil	330,890	Brazil	21,048
			Peru	111,698	Mexico	6,989
			Canada	82,469	Canada	6,250
			Mexico	62,527	Peru	3,244
Europe	1,793,907	168,193	Russia	326,448	United Kingdom	36,393
			United Kingdom	254,195	Italy	32,616
			Spain	234,824	Spain	28,628
			Italy	228,658	France	28,289
			Germany	177,850	Belgium	9,212
Oceania	8,540	129	Australia	7,095	Australia	101
			New Zealand	1,154	New Zealand	21
			Guam	165	Guam	5
			FP	60	NMI	2
			NMI	22	FP	0

*FP, French Polynesia; NMI, Northern Mariana Islands.*

As can be seen in Table 1, Spain and Mexico are among the most affected countries, with Spain ranking third in Europe in terms of deaths reported by COVID-19, and Mexico ranking third in terms of deaths reported by COVID-19 in the Americas.

On one hand, in Spain, the situation has been particularly complicated. As of May 23, 2020, there have been 234,824 confirmed cases in Spain, and 28,628 have died according to official data from the European Centre for Disease Prevention and Control (ECDC) (European Centre for Disease Prevention and Control, 2020d). The first positive diagnosis was confirmed on January 31, 2020 (Linde, 2020), while the first death occurred on February 13 in the city of Valencia (Caparrós, 2020). In view of the rapid spread of the virus, since March 14, the Spanish government has decreed a state of alarm (Boletín Oficial del Estado, 2020), restricting the mobility of citizens to certain cases, such as the purchase of food and medicines or visits to medical centers or the workplace. All face-to-face teaching activities have been interrupted in Spain since March 16, after the state of alarm was decreed (Boletín Oficial del Estado, 2020), although some communities such as Madrid or La Rioja imposed this measure on their schools a few days earlier. In total, some 10 million students from all educational stages are currently following their academic year at a distance (Faro de Vigo, 2020). A large number of teachers took on the tasks of distance teaching without being previously trained for them, nor having specific resources for all this in many cases.

This whole situation of stress caused by the pandemic, together with changes in the usual working conditions, could negatively affect not only the psychosocial risks of teachers but also its main consequences such as burnout.

On the other hand, in the case of Mexico, the first positive diagnosis was confirmed on February 27 in Mexico City (British Broadcasting Corporation, 2020), almost a month after in Spain. On March 30, a “health emergency due to force majeure” was declared as a result of the evolution of confirmed cases and deaths from the disease in the country, which led to the implementation of additional actions for its prevention and control (Secretaría de Salud, Gobierno de México, 2020). Currently, there have been 62,527 cases, of which 42,725 have been discharged and 6,989 have died, according to official data from the Mexican government and ECDC (European Centre for Disease Prevention and Control, 2020d; Secretaría de Salud, Gobierno de México, 2020).

Despite the fact that Spain is one of the countries most affected by the pandemic in Europe, and Mexico in America, we could say that the phase in which both countries are in this sense is different, since at the moment, it seems that Spain has reached its peak long before Mexico, with the former being in a phase of decreasing new cases, while the latter seems to be in the middle of a phase of increasing new cases. Of the total number of cases of COVID-19 in Spain (234,824), only 10,863 have been recorded in the last 14 days, while of the total number of cases in Mexico (62,527), 31,005 have been recorded in the last 14 days, i.e., the figure has practically doubled in the last 2 weeks (as of May 23, 2020) (European Centre for Disease Prevention and Control, 2020d).

The pandemic caused by COVID-19 generates a series of impacts in all spheres of society, posing a challenge in practically all areas. During the pandemic, the population has had to adapt to a number of situations where uncertainty, fear, and, in many cases, pain have been present. These effects may translate into a range of emotional reactions (such as distress or psychiatric conditions), unhealthy behaviors (such as excessive substance use), and non-compliance with public health directives (such as home confinement and vaccination) in people who contract the disease and in the general population (Pfefferbaum, 2020). One of the many sectors that has had to adapt to this new situation and the demands that it entails is that of education. Teachers at all levels of education have tried to maintain their fundamental role and continue to carry out their teaching duties, despite the uncertainty of the situation, the difficulties related to their own health and that of their loved ones, the lack of resources (material and knowledge), and the huge amount of extra work that adaptation to teach from home entails, including helping their students to cope to this situation. In particular, non-university teachers (primary and secondary education), aware of the fundamental importance of learning for the development and future school performance of their students, have faced this situation by providing, in most cases, distance education, even if resources were often not available and uncertainty about the situation has always been present. In this context, the factors that are normally important for the good professional performance and well-being of teachers become even more relevant. Among them are the so-called psychosocial risks.

### Psychosocial Risks

Cox and Griffiths (2005) define psychosocial risks at work as aspects regarding work design as well as the social, organizational, and management contexts of work that could potentially cause physical or psychological harm. Psychosocial risks and work-related stress are among the most challenging issues in occupational safety and health, impacting significantly on the health of individuals, organizations, and national economies (Bailey et al., 2015; Bergh et al., 2018). Psychosocial risks arise from poor work design, organization, and management, as well as a poor social context of work, and they may result in negative psychological, physical, and social outcomes such as work-related stress, burnout, or depression (European Centre for Disease Prevention and Control, 2020d). More specifically, psychosocial risks have been shown to be related to low job satisfaction (Guadix et al., 2015), health problems (Bergh et al., 2018), work accidents (Fornell et al., 2018), work-related stress (Junne et al., 2018), and burnout (Maslach et al., 2001; Elshaer et al., 2018). Psychosocial risks are closely related to work-related stress, which has been associated with a reduction in social interaction and the ability to concentrate at work, an increase in physiological pain and cardiovascular problems, and a higher incidence of mental illness such as depression and anxiety (Nielsen et al., 2020). In this same vein, the right management of psychosocial risk helps to prevent accidents and absenteeism (Maslach, 2017), to increase productivity (Bakker and Demerouti, 2017; Bakker and Wang, 2019) and to promote well-being at the workplace (Hammer et al., 2019).

Among the different theoretical models that exist to explain the appearance of occupational stress, Karasek (1979) model is the one with the most theoretical and empirical support and it is the one that currently has the most influence and attention. It explains work-related stress according to the imbalance between psychological demands at work (e.g., workload, role conflicts, interpersonal conflicts, job insecurity,…) and the control level or resources that the employee has. According to this model, the employee health or well-being depends on the balance of the work demands and the resources that the employees have. When the demands are higher than the resources, it can create a feeling of work-related stress in the employee. In addition, the chronic work-related stress can cause burnout syndrome, being able to appear as several physical or psychosomatic symptomatology. Thus, an excess of demands will produce a negative consequence in the employee, as higher burnout; however, having enough resources benefits the employee, decreasing the probability of having higher burnout (Hatch et al., 2018).

Among the different psychosocial risks, the following stand out because of their importance in relation to the crisis situation and the theoretical reference model: (a) Role conflict: this is the situation in which a worker cannot simultaneously satisfy the contradictory role expectations in which he or she is involved. There is role conflict when a worker is being given work tasks without enough resources to complete them and receiving contradictory requests from different people. Previous research has shown that problematic levels of distress were 53% more likely for workers reporting role conflict (Johannessen et al., 2013). Having to teach from your own home often can bring some role conflict, since familiar conciliation might get more challenging for those teachers who also have to perform other roles, such as being parents, partners, and caregivers in general. (b) Lack of organizational justice: Lack of organizational justice refers to the extent to which employees perceive they are treated unfairly in their workplace and the perception of the absence of reciprocity in social exchanges (Moorman, 1991; Kobayashi and Kondo, 2019). Low organizational justice is known to be a potential risk factor for poor physical and psychological health among employees (Fujishiro and Heaney, 2009; Kobayashi and Kondo, 2019). (c) Workload: It assesses quantitative and qualitative workload. Quantitative workload refers to the amount of activities to be performed in a given period of time, while qualitative workload refers to the difficulty of the task and the volume of information to be processed in relation to the time available (Gil-Monte, 2016). A high workload has been associated with low levels of well-being and higher risks of health problems (Pace et al., 2019). In general, the adaptation to the current pandemic situation requires an extra load of work that teachers (and family and students) have to deal with. (d) Interpersonal conflicts: It assesses the frequency with which workers perceive conflicts coming from the school management, colleagues, students, or relatives of the students. Interpersonal conflicts have been associated to health problems, particularly to depression (Kubik et al., 2018). In the context of the COVID-19 pandemic, uncertainty has often made it difficult to reach an agreement between school, families, students, and teachers about the best way to proceed, which new measures to take in order to adapt, and for how long this measures should be maintained. (e) Emotional work: It refers to the effort, planning, and control necessary to express the organizationally desirable emotions during interpersonal transactions (Morris and Feldman, 1996; Ortiz et al., 2012). Previous research has shown that problematic levels of distress were 38% more likely for workers reporting high emotional work (Johannessen et al., 2013). In the context of a pandemic, an extra burden of negative emotions in teachers (such as worry, uncertainty, and fear) would be expected. (f) Job insecurity: the perceived threat of losing one’s current job in the near future (Heaney et al., 1994), or that the employer did not comply with his or her obligations or promises (breach of psychological contract) (Vander Elst et al., 2016), can have equally serious consequences as actual job loss (De Witte, 1999). Particularly, job insecurity is considered a stressor that affects negatively the physical, psychological, and social health of the employee (Cheng and Chan, 2008; De Witte et al., 2015; De Witte et al., 2016; Selenko et al., 2017).

On the other hand, as the Karasek model points out, one of the most important resources to cope with psychosocial risk factors is the *Social support at work*. Social support at work is defined as the social climate in the work context involving the relationship between the supervisor and coworkers (Karasek and Theorell, 1990). It involves both social–emotional and instrumental support. The former refers to the degree of social and emotional integration between coworkers and the supervisor, while instrumental support refers to the collaboration between coworkers and the supervisor to carry out work tasks (Martín-Arribas, 2007).

An important potential source of social support is the emotional support of family, friends, and colleagues, which is particularly difficult to have on a confinement situation such as that originated by the COVID-19 pandemic. Social support and resilience protect individuals from threats to their mental and physical health by reducing or balancing the negative effects of the stressful events they experience in life (Woodhead et al., 2016; Sun et al., 2017).

As previously stated, a large number of demands and low resources produce a series of negative consequences for workers (Karasek, 1979), of which psychosomatic health problems and burnout syndrome are the most important due to their prevalence and associated consequences.

#### Psychosomatic Health Problems

The term psychosomatic refers to all those alterations in which mental processes influence areas of the organism (Montiel et al., 2016). Among the most common are various types of symptoms affecting multiple organs and systems. Examples of these are back pain, tension headaches, sleep problems, chronic fatigue, heartburn, tension diarrhea, or heart palpitations (Jaradat et al., 2016).

#### Burnout Syndrome

Burnout syndrome is defined as a prolonged response to chronic emotional and interpersonal stressors at work and is defined by the three dimensions of burnout, cynicism, and inefficiency (Maslach et al., 2001).

The prevalence of burnout in education oscillates between 11 and 35.5% depending on the country and the study considered (Ratto et al., 2015; Villaverde et al., 2019). A Eurofound study (Aumayr-Pintar et al., 2020) showed that, in Portugal, 15% of educational professionals had a moderate risk of burnout, and the employees with burnout syndrome increased from 8 to 15% between 2008 and 2013. In addition, their study (King et al., 2018) with school counselors in Australia found that 45% of the sample experiences burnout.

Given this prevalence in recent years, the study of burnout in the education sector has become increasingly important (Kim and Burić, 2019; McLean et al., 2019a; Schonfeld et al., 2019). Most researches pointed out the importance of burnout on teachers (Kaur and Singh, 2014; Yerdelen et al., 2016; Schonfeld et al., 2019), considering it as a risk for teachers that can negatively affect effective teaching (Travers, 2017), their interaction with their students (Travers, 2017), and their motivation for the job (McLean et al., 2019b), resulting in absenteeism (Makhdoom et al., 2019), depression (Martínez-Monteagudo et al., 2019), insomnia (Gu et al., 2020), or a decrease in the capacity to give support to the students (Zapf et al., 1999; Jennings and Greenberg, 2009).

Despite the impact of pandemics on the health and well-being of citizens, and more specifically of workers, and their clear influence on working conditions, or more specifically on their psychosocial risks, there are hardly any studies that have addressed the effect of a pandemic on psychosocial risks. There are even fewer studies comparing these types of factors during a pandemic in Spanish-speaking countries. Although there are studies carried out within the framework of different crises, allowing for contextualization of stress situations, these do not focus on the specific case of a pandemic like the one we are facing due to COVID-19. This situation is even more limited if we consider the impact on teachers. Likewise, the few studies traditionally available have been carried out retrospectively, ignoring their perception of the pandemic, as well as the associated psychosocial risks during the times of greatest severity.

After conducting a review of the literature, we were unable to observe any studies focused on teachers that analyzed the psychosocial risks of this group and their perception of the pandemic comparing two Spanish-speaking countries at different phases or moments of the pandemic. Therefore, the study presented here aims to fill this gap in the literature by offering a first approach to the perception of COVID-19 by teachers and its relationship with psychosocial risks, comparing data from Spain and Mexico.

### Aims

The main aim of this study was to analyze the perception of non-university teachers regarding measures and resources implemented by institutions and governments and its impact on their daily work. Also, to analyze the psychosocial risks of these professionals and its relation to the sanitary emergency caused by COVID-19 comparing two Spanish-speaking countries, Spain and Mexico, at a moment where the two countries were at different phases of the pandemic.

## Materials and Methods

### Design, Procedure, and Participants

Data were collected from a sample of 421 non-university teachers (80.2% women and 19.8% men; 56.3% from Mexico and 43.7% from Spain) aged 24–60 years (*M* = 39.32, *SD* = 10.21) *via* a self-completed questionnaire during the COVID-19 pandemic from March to April 2020. From Spain, participants were aged 24–60 (*M* = 40.17, *SD* = 8.46), 71.1% of whom were women and 28.9% were men. From Mexico, participants were aged 20–64 (*M* = 38.72, *SD* = 811.28), 86.6% of whom were women and 13.4% were men. At the beginning of the study, the research team contacted different associations and institutions of education in order to reach non-university teachers and invite them *via* e-mail to participate in the study. In the online invitation, teachers were informed about the purpose of the study and also about how their anonymity and confidentiality were guaranteed. The time cost of completing the questionnaire was 35 min.

The eligibility criteria for participants were as follows.

Inclusion criteria:

(a) To be a teacher in an institution other than university.
(b) To be actively working during the moment of assessment.
(c) To have signed the informed consent document and confidentiality agreement within the framework of the principles of the Declaration of Helsinki.

### Outcome Measures

The research included the variables and measurement instruments:

#### Psychosocial Risks

Different questionnaires were used to measure demands, resources, and consequences: The UNIPSICO Battery (Gil-Monte, 2016), the Burnout Assessment Tool (BAT) (Schaufeli et al., 2019), and The Job Insecurity Scale (Vander Elst et al., 2014).

The demand factors include:

##### Role conflict

Taken from UNIPSICO Battery (Gil-Monte, 2016). Role conflict is the situation in which a worker cannot simultaneously satisfy the contradictory role expectations in which he or she is involved. The scale is composed of five items (e.g., “I receive incompatible demands from two or more people”). Participants are asked to score the frequency with which they have experienced the situation described in each statement on a Likert-type scale from 0 to 4 (0 = Never; 4 = Very frequently: every day), with higher scores indicating higher levels of Role conflict (scores above 1.6 are considered high, whereas scores equal to or below 0.81 are considered low). The scale has obtained adequate psychometric properties in previous studies and in the present research (total sample: α = 0.84; Mexico: α = 0.80; Spain: α = 0.84).

##### Lack of organizational justice

Extracted from UNIPSICO Battery (Gil-Monte, 2016). Lack of organizational justice is defined as the perception of the absence of reciprocity in social exchanges. The scale is made up of five items (e.g., “I give up my skin at work compared to what I receive in return”). The response format is on a Likert-type scale from 0 to 4 (0 = Never; 4 = Very frequently: every day), with higher scores indicating higher lack of organizational justice (scores above 2.4 are considered high, whereas scores equal to or below 1.6 are considered low). The scale has obtained adequate psychometric properties in previous studies and in the present research (total sample: α = 0.83; Mexico: α = 0.74; Spain: α = 0.89).

##### Workload

Taken from UNIPSICO Battery (Gil-Monte, 2016). It assesses quantitative and qualitative workload on a Likert-type scale from 0 to 4 (0 = Never; 4 = Very frequently: every day). Quantitative workload refers to the amount of activities to be performed in a given period of time, while qualitative workload refers to the difficulty of the task and the volume of information to be processed in relation to the time available. It consists of six items, three quantitative (e.g., “Is it possible for you to work at a relaxed pace?”) and three qualitative (e.g., “When you are working, do you encounter particularly hard situations?”), with higher scores indicating higher Workload (scores above 2.17 are considered high, whereas scores equal to or below 1.51 are considered low). The scale has obtained adequate psychometric properties in previous studies and in the present research (total sample: α = 0.76; Mexico: α = 0.62; Spain: α = 0.80).

##### Interpersonal conflicts

Extracted from UNIPSICO Battery (Gil-Monte, 2016). It assesses the frequency (0 = Never; 4 = Very frequently: every day) that workers perceive conflicts coming from the hospital management, colleagues, patients, and relatives of the patient. The scale consists of six items (e.g., “How often do you have conflicts with your colleagues?”), with higher scores indicating higher Interpersonal conflicts (scores above 1 are considered high, whereas scores equal to or below 0.6 are considered low). The scale has obtained adequate psychometric properties in previous studies and in the present research (total sample: α = 0.60; Mexico: α = 0.58; Spain: α = 0.57).

##### Job insecurity

It was measured using the Job Insecurity Scale (Vander Elst et al., 2014). It consists of five items (e.g., “I feel insecure about the future of my job”) designed to measure quantitative job insecurity (i.e., insecurity to lose the job as such). Respondents were asked to rate these items on a 5-point Likert-type scale, ranging from 1 (“strongly disagree”) to 5 (“strongly agree”), with higher scores indicating higher levels of job insecurity. The scale has obtained adequate psychometric properties in previous studies and in the present research (total sample: α = 0.87; Mexico: α = 0.76; Spain: α = 0.91).

The resource factors include:

##### Social support at work

Extracted from UNIPSICO Battery (Gil-Monte, 2016). This is defined as the availability of help from other people. It evaluates the social support offered by your head of studies, the management of the center, and by your colleagues, in all cases in the form of emotional support and technical support. It consists of six items (e.g., “How often do your colleagues help you when problems arise at work?”). This was answered on a 4-pont Likert-type scale (0 = Never; 4 = Very frequently: every day), with higher scores indicating higher Social support at work (scores above 2.83 are considered high, whereas scores equal to or below 2 are considered low). The scale has obtained adequate psychometric properties in previous studies and in the present research (total sample: α = 0.88; Mexico: α = 0.88; Spain: α = 0.89).

The consequence factors include:

##### Psychosomatic problems

Included in the UNIPSICO battery (Gil-Monte, 2016). It assesses the frequency of occurrence of psychosomatic problems related to the perception of sources of stress at work. It consists of nine items related to different systems of the organism (e.g., “Have you been worried that, without making any effort, your breathing would be cut off?”). It was answered on a 4-pont Likert-type scale (0 = Never; 4 = Very frequently: every day), with higher scores indicating higher Psychosomatic problems (scores above 1.67 are considered high, whereas scores equal to or below 0.89 are considered low). The scale has obtained adequate psychometric properties in previous studies and in the present research (total sample: α = 0.90; Mexico: α = 0.90; Spain: α = 0.90).

##### Burnout

It was assessed using the reduced version of the BAT (Schaufeli et al., 2019). It consists of 12 items that evaluate four scales: (European Centre for Disease Prevention and Control, 2020c) exhaustion (e.g., “At work, I feel mentally exhausted”), mental distance (e.g., “At work, I have trouble staying focused”), emotional impairment (e.g., “I don’t recognize myself in the way I react emotionally at work”), and cognitive impairment (e.g., “I make mistakes in my work because I have my mind on other things”). Participants are asked to score the frequency that they have experienced the situation described in each statement on a Likert-type scale from 0 to 4 (0 = Never; 4 = Very frequently: every day), with higher scores indicating higher levels of Burnout. The instrument has obtained adequate psychometric properties in previous studies and in the present research (total sample: α = 0.91; Mexico: α = 0.89; Spain: α = 0.91).

#### Coronavirus Disease 2019-Related Measures

This is an *ad hoc* questionnaire of 13 items constructed to measure different aspects related to the health emergency caused by the COVID-19. The aspects considered are: Available resources (provided by the health center, regional government, and national government, e.g., “I feel that my center has put sufficient resources to deal with COVID-19 in my daily work”), information {provided by the health center, regional government, and national government, e.g., “I consider that from the regional government [e.g., state of Sinaloa (Mexico)/or Autonomous Community (Spain)] I have been given enough information to deal with COVID-19 in my daily work”}, measures (taken by the health center, regional government, and national government, e.g., “I believe that sufficient measures have been taken by the national government to address COVID-19 in my daily work”), and impact on work (workload, labor conflicts, work-related stress, and work-related concerns and fears, e.g., “The COVID-19 has increased my workload”). The subjects score on a Likert-type scale his or her level of agreement or disagreement with the statements (1 = totally disagree, 5 = totally agree). Scores range from 1 to 5, with higher levels indicating greater satisfaction with the resources available, information, and measures taken, as well as higher levels of impact on work. The scale has obtained adequate psychometric properties (total sample: available resources α = 0.90, information α = 0.94, measures α = 0.94, impact on work α = 0.78; Mexico: available resources α = 0.86, information α = 0.93, measures α = 0.91, impact on work α = 0.78; Spain: available resources α = 0.87, information α = 0.89, measures α = 0.90, impact on work α = 0.78).

### Data Analyses

A descriptive statistical analysis was performed for all study variables, as well as correlations and mean comparison analysis. All analyses were carried out using the statistical package SPSS (Statistical Package for the Social Sciences, Version 25, Armonk, NY, United States: IBM Corp.).

## Results

### Descriptive Analysis

#### Sociodemographic

From the total sample of 421 non-university teachers, 237 were from Mexico and 184 from Spain. The great majority worked in a public institute (84.8%); 32% taught in kindergarten, 39.5% in primary school, and 28.5% in high school. The educational level of the teachers was 65.3% university degree, 23.9% master, and 10.8% doctorate. From the participants, 7.8% had a temporary contract, whereas 92.2% had a permanent contract.

#### Psychosocial Risks

As it can be seen in Table 2, regarding psychosocial risks, that teachers in Spain present medium levels on all of the psychosocial risks, whereas teachers in Mexico present medium levels on lack of organizational justices and of social support and low levels on the rest of psychosocial risks.

**TABLE 2:** Descriptive data on psychosocial risks of teachers in Spain and Mexico.

		Role conflict	Lack of organizational justice	Workload	Interpersonal conflicts	Job insecurity	Psychosomatic problems	Burnout	Social support
Spain	Mean	1.07	2.04	1.76	0.72	1.90	0.92	1.14	2.40
	*SD*	0.69	1.05	0.76	0.61	1.14	0.60	0.67	1.00
	Range	0–4	0–4	0–4	0–4	1–5	0–4	0–4	0–4
	Level of risk	Medium	Medium	Medium	Medium	–	Medium	–	Medium
Mexico	Mean	0.66	1.65	1.17	0.37	1.42	0.76	0.68	2.50
	*SD*	0.57	0.82	0.57	0.47	0.71	0.56	0.51	0.94
	Range	0–4	0–3.83	0–4	0–4	1–5	0–4	0–4	0–4
	Level of risk	Low	Medium	Low	Low	–	Low	–	Medium

*SD, standard deviation; –, no available scale ranges for severity of this variables in teachers’ population.*

#### Coronavirus Disease 2019-Related Measures

As it can be seen in Table 3, during the pandemic, teachers in Spain rated the resources, information available, and measures taken by the government and the hospital below the mean value of the answer scale, which points to a tendency to consider resources, information, and measures as insufficient. The highest scores from teachers in Spain are regarding the impact of COVID-19 on their jobs, although the scores in this case are also below the medium value of the answer scale. Teachers in Mexico, on the other hand, rated the resources and information available and the measures taken by government and the hospital above the mean value of the answer scale, which points to a tendency to consider resources, information, and measures as sufficient. The lowest score for teachers in Mexico is the impact of COVID-19 on their jobs.

**TABLE 3:** Descriptive data on COVID-19-related measures on teachers in Spain and Mexico.

		Resources	Information	Measures	Impact
Spain	Mean	2.10	2.31	2.11	2.59
	*SD*	1.12	1.18	1.15	1.11
	Range	1–5	1–5	1–5	1–5
Mexico	Mean	3.32	3.84	3.65	2.35
	*SD*	1.32	1.23	1.20	1.07
	Range	1–5	1–5	1–5	1–5

*SD, standard deviation.*

### Comparison of Mean

Analysis of the mean comparison among the variables of the study was carried out between data from teachers in Spain and Mexico (Table 4).

**TABLE 4:** Means, SDs, effect sizes, mean comparison.

	Teachers in Spain	Teachers in Mexico	Cohen’s *d*	Test *t*	
	*M (SD)*	*M (SD)*		*t*	*p*
Role conflict	1.07(0.69)	0.66(0.57)	0.65	–6.37	0.000
Lack of organizational justice	2.04(1.05)	1.65(0.82)	0.48	–4.15	0.000
Workload	1.76(0.76)	1.17(0.57)	0.88	–8.81	0.000
Interpersonal conflicts	0.72(0.61)	0.37(0.47)	0.64	–6.42	0.000
Job insecurity	1.90(1.14)	1.42(0.71)	0.51	–4.97	0.000
Psychosomatic problems	0.92(0.60)	0.76(0.56)	0.28	–2.77	0.006
Burnout	1.14(0.67)	0.68(0.51)	0.77	–7.71	0.000
Social support	2.40(1.00)	2.50(0.94)	–	1.10	0.274
COVID19 resources	2.10(1.12)	3.32(1.32)	0.99	10.15	0.000
COVID19 information	2.31(1.18)	3.84(1.23)	1.27	12.78	0.000
COVID19 measures	2.11(1.15)	3.65(1.20)	1.31	13.24	0.000
COVID19 impact	2.59(1.11)	2.35(1.07)	0.22	–3.06	0.024

*M, mean; SD, standard deviation; Cohen’s d, effect size; p, probability.*

In general, it seems that the pandemic has a greater effect in the case of Spain, since there are statistically significant differences in all dimensions except social support, with higher levels of risk and consequences in the Spanish case. Likewise, there is greater satisfaction with the available information, resources, and measures in the Mexican case than in the Spanish case, and finally, there seems to be a greater impact of the pandemic on the work and life of teachers in the Spanish case in comparison with the Mexican case.

### Analysis of Relations

The results of the correlation analysis among the variables are shown in Table 5. As it can be seen, almost all the variables are very strongly related. The only correlation that is not statistically significant is between the Impact of COVID-19 in the workplace and Social support.

**TABLE 5:** Correlations among all the variables of the study.

	RC	LOJ	WL	IC	JI	PP	B	SS	R	INF	M	IMP
Role conflict	1											
Lack of organizational justice	0.46**	1										
Workload	0.71**	0.53**	1									
Interpersonal conflicts	0.65**	0.32**	0.57**	1								
Job insecurity	0.21**	0.16**	0.22**	0.14**	1							
Psychosomatic problems	0.61**	0.42**	0.64**	0.54**	0.15**	1						
Burnout	0.71**	0.46**	0.76**	0.55**	0.23**	0.68**	1					
Social support	−0.35**	−0.27**	−0.22**	−0.27**	−0.18**	−0.26**	−0.31**	1				
COVID-19: resources	−0.36**	−0.31**	−0.38**	−0.26**	−0.17**	−0.33**	−0.40**	0.25**	1			
Information	−0.41**	−0.31**	−0.41**	−0.32**	−0.16**	−0.35**	−0.44**	0.25**	0.80**	1		
Measures	−0.36**	−0.31**	−0.41**	−0.29**	−0.20**	−0.31**	−0.41**	0.24**	0.86**	0.85**	1	
Impact	0.27**	0.23**	0.31**	0.24**	0.11*	0.34**	0.32**	–0.05	−0.20**	−0.21**	−0.19**	1

RC, role conflict; LOJ, lack of organizational justice; WL, workload; IC, interpersonal conflicts; JI, job insecurity; PP, psychosomatic problems; B, burnout; SS, social support; R, resources; M, measures; INF, information; IMP, impact; *p < 0.05, **p < 0.01.

When focusing on correlations among the variables in teachers from Spain and Mexico separately, the situation slightly changes (Table 6). In the case of teachers in Spain, many of the psychosocial risks correlate between them, except for Job insecurity that does not appear related to any of the resting variables. Also, from the COVID-19-related measures, Information and Measures are related with a higher number of psychosocial risks, whereas Impact is less related to the rest of the variables (psychosocial risks as well as the resting COVID-19-related measures). On the other hand, in the case of teachers in Mexico, variables are also very strongly related between them, although Lack of organizational justice and Job insecurity are less related to the rest of the variables. Also, in contrast with the case of Spain, Impact appears related with the rest of the COVID-19-related measures.

**TABLE 6:** Correlations among all the variables of study in Spain (upper diagonal) and in Mexico (lower diagonal).

	RC	LOJ	WL	IC	JI	PP	B	SS	R	INF	M	IMP
Role conflict	1	0.53**	0.72**	0.67**	0.11	0.65**	0.71**	−0.37**	−0.19**	−0.29**	−0.20**	0.28**
Lack of organizational justice	0.31**	1	0.55**	0.37**	0.08	0.51**	0.49**	−0.41**	−0.31**	−0.38**	−0.32**	0.20**
Workload	0.62**	0.43**	1	0.58**	0.11	0.69**	0.73**	−0.26**	−0.19**	−0.23**	−0.20**	0.29**
Interpersonal conflicts	0.56**	0.15*	0.41**	1	0.04	0.61**	0.60**	−0.37**	−0.16*	−0.21**	–0.14	0.23**
Job insecurity	0.19**	0.16*	0.15*	0.11	1	0.09	0.10	–0.13	–0.03	0.05	–0.04	0.02
Psychosomatic problems	0.56**	0.28**	0.60**	0.44**	0.18**	1	0.70**	−0.30**	−0.25**	−0.30**	−0.24**	0.43**
Burnout	0.64**	0.32**	0.59**	0.37**	0.24**	0.69**	1	−0.35**	−0.21**	−0.28**	−0.21**	0.32**
Social support	−0.33**	–0.12	−0.18**	−0.18**	−0.24**	−0.21**	−0.27**	1	0.27**	0.28**	0.25**	–0.03
COVID-19:												
Resources	−0.34**	−0.21**	−0.31**	−0.14*	–0.13	−0.35**	−−0.36**	0.24**	1	0.79**	0.92**	–0.13
Information	−0.33**	–0.12	−0.29**	−0.19**	−0.13*	−0.35**	−0.37**	0.25**	0.71**	1	0.79**	−0.18*
Measures	−0.29**	−0.18**	−0.29**	−0.17**	–0.13	−0.33**	−0.34**	0.25**	0.78**	0.80**	1	–0.13
Impact	0.22**	0.23**	0.30**	0.21**	0.17**	0.24**	0.29**	–0.05	−0.19**	−0.16*	−0.16*	1

RC, role conflict; LOJ, lack of organizational justice; WL, workload; IC, interpersonal conflicts; JI, job insecurity; PP, psychosomatic problems; B, burnout; SS, social support; R, resources; M, measures; INF, information; IMP, impact; *p < 0.05, **p < 0.01.

## Discussion and Conclusion

The current crisis caused by the coronavirus is a challenge not only in the health field but also in all spheres of society. In this context, professionals at all levels have had to adapt to new working conditions, in addition to dealing with the pandemic in their personal lives and as members of the community. Among them, teachers of preschool and primary and secondary education have had to assume their important role in the best possible way, with limited means and resources and with the uncertainty of the moment and with the enormous responsibility that comes with educating and training children and adolescents, helping them to cope with the crisis and often providing relief as much as possible with homework and how to take school home (Boletín Oficial del Estado, 2020; Faro de Vigo, 2020). Considering that teachers are vulnerable to burnout and job stress (Zapf et al., 1999; Jennings and Greenberg, 2009; Kaur and Singh, 2014; Yerdelen et al., 2016; Travers, 2017; Makhdoom et al., 2019; Martínez-Monteagudo et al., 2019; McLean et al., 2019b; Schonfeld et al., 2019; Gu et al., 2020), and therefore the negative consequences these can have on their health and professional performance (Bergh et al., 2018; Fornell et al., 2018; Junne et al., 2018; European Centre for Disease Prevention and Control, 2020d), it is essential to study how psychosocial risks affect this group at a time of such vulnerability and general demand as the present. The literature on social risks to teachers in a pandemic context is extremely limited; however, it is critical to study the extent to which factors related to teachers’ well-being may be affected during a health crisis such as the current one in order to ensure the well-being of teachers and, in turn, the children and adolescents in their care.

This study has sought to explore the extent to which teachers are affected by psychosocial risks during the pandemic and how these risks relate to teachers’ perceptions of the pandemic in terms of resources, measures, information, and impact. At the same time, it compares data of teachers in Mexico with data of teachers in Spain, two countries heavily affected by the pandemic and yet at very different stages of its development: Spain in the midst of a drop in cases, Mexico in the midst of a rise (European Centre for Disease Prevention and Control, 2020d).

At the time of collecting the data, the coronavirus crisis was at its peak in Spain, while in Mexico, it was in a more initial phase. This facilitates the interpretation of some of the data found.

The main results of the study show, on the one hand, that teachers in Spain as well as teachers in Mexico inform about perceiving lack of organizational justice during the pandemic but, at the same time, to perceive social support. Teachers in Spain, however, also inform about role conflict, workload, interpersonal conflict, psychosomatic problems, and burnout. These data go in line with previous literature about the social risks that teachers are exposed to (Zapf et al., 1999; Jennings and Greenberg, 2009; Kaur and Singh, 2014; Yerdelen et al., 2016; Travers, 2017; Makhdoom et al., 2019; Martínez-Monteagudo et al., 2019; McLean et al., 2019b; Schonfeld et al., 2019; Gu et al., 2020). Regarding resources and information available about COVID-19, the impact of COVID-19 on their jobs, as well as measures taken by responsible entities (national and regional government, as well as work center), data from teachers in Spain point to a perception of insufficient resources, information, and measures and to a perception of a moderate–high impact of COVID-19 on their jobs. Regarding teachers in Mexico, data point to a perception of sufficient resources, information, and measures taken by responsible entities, as well as to a perception of a moderate impact of COVID-19 on their jobs.

When specifically comparing data from teachers in Spain and Mexico, the results highlight a difference between teachers in both countries: Spanish teachers present more role conflict, lack of organizational justice, workload, interpersonal conflicts, job insecurity, psychosomatic problems, and burnout than teachers in Mexico. At the same time, teachers in Spain inform about less resources, information, and measures than teachers in Mexico, but also about a bigger impact of COVID-19 on their jobs, than teachers in Mexico. The fact that teachers in Spain are more affected by psychosocial risks during the pandemic and are more burned out by work could be due, on the one hand, to the phase of the pandemic at the time of data collection, as the pandemic situation was more severe in Spain at the time that the study was conducted. However, it could also be due to the fact that teachers in Spain perceive fewer resources, information, and measures taken by responsible institutions, which could in turn worsen some of the psychosocial risks and even be a direct risk factor for burnout. Specifically, in terms of the relationship between psychosocial risk factors and COVID-19-related measures, these appear to be closely related, although it is true that in the case of teachers in Spain, the relationship between COVID-19-related measures and social risks is clearer than in the case of teachers in Mexico. Of the COVID-19-related measures, the least related to psychosocial risks is the impact of COVID-19 on work, while of the psychosocial risks, the least related to the rest of the psychosocial risks and to COVID-19-related measures is Job insecurity. These data indicate that teachers’ perception of the measures taken by the responsible entities, as well as the perception of sufficient information and resources, could influence the psychosocial risks to which these professionals are exposed. As mentioned above, some of the differences are due, on the one hand, to the phase of the pandemic in which both countries were and, on the other hand, to the perception of resources by teachers to face the pandemic and the challenges it poses in their professional life.

One of the main limitations of this study is that it presents an analysis of relationships between variables that does not allow for the establishment of causal relationships between them. Furthermore, it is a cross-sectional study that does not allow for observing the evolution of the data as the pandemic caused by COVID-19 progresses. Future studies could make new measurements of the variables when the different phases of the pandemic have passed, which would allow the comparison of the variables taking into account the evolution of the health crisis, as well as the evolution of the psychosocial risks of teachers and the possible development of pathologies that, based on the scientific literature, have been related to the burnout and psychosocial risks described here.

Despite its limitations, this study shows data collected in a context never before seen, where data on psychosocial risks are not collected *a posteriori* but in the midst of a pandemic crisis. Our data speak of a greater general attrition of teachers in Spain, which indicates that the pandemic may indeed be related to greater sources of stress and psychosocial risks. At the same time, data from the present study underline the importance of the perception of resources by professionals, which could prevent to some extent the burnout and the possible alterations associated with it.

It is difficult to carry out this type of study in these contexts for a number of reasons, but we believe that it is important to have data to support the fact that the measures taken by the responsible entities and the provision of information affect teachers not only directly but also indirectly by making them more vulnerable to psychosocial risks that could affect their health and professional performance, thus affecting students as well. If this is important in any context, it becomes even more important in a context where the emotional toll on society is more evident than ever.

Some of the main practical applications of this research would be to know the psychosocial risks during a pandemic in non-university teachers to discover the perception of resources, information, and measures adopted by the different public and private entities to deal with COVID-19, as well as to know the impact that this perception has had on the daily work of non-university teachers. These results can help make a difference between building resilience and developing burnout. Any data that can clarify the relationships between the variables will be data that will benefit teachers, their students, and society in general. The results obtained in the present study allow to advance and consolidate the research on psychosocial risks during a pandemic while enabling the development of policies for action to improve teachers’ coping with a pandemic and occupational health, which in turn will impact the outcomes of their work and society as a whole.

## Data Availability Statement

The raw data supporting the conclusions of this article will be made available by the authors, without undue reservation, to any qualified researcher.

## Ethics Statement

Ethical review and approval was not required for the study on human participants in accordance with the local legislation and institutional requirements. The patients/participants provided their written informed consent to participate in this study.

## Author Contributions

VP-G made a substantial contribution to the concept and design of the work, as well as on analysis and interpretation of data, drafted the article and revised it critically for important intellectual content, approved the version to be published, and participated sufficiently in the work to take public responsibility for appropriate portions of the content. MG-D made a substantial contribution to the concept of the work and acquisition of data, revised the article, approved the version to be published, and participated sufficiently in the work to take public responsibility for appropriate portions of the content. AS-R made a substantial contribution to the concept or design of the work and on interpretation of data, drafted the article and revised it critically for important intellectual content, approved the version to be published, and participated sufficiently in the work to take public responsibility for appropriate portions of the content. LD-R made a substantial contribution to the design of the work and the acquisition of data, revised the article critically for important intellectual content, approved the version to be published, and participated sufficiently in the work to take public responsibility for appropriate portions of the content. DN-M made a substantial contribution to the concept and design of the work and acquisition of data, drafted the article and revised it critically for important intellectual content, approved the version to be published, and participated sufficiently in the work to take public responsibility for appropriate portions of the content. All authors contributed to the article and approved the submitted version.

## Conflict of Interest

The authors declare that the research was conducted in the absence of any commercial or financial relationships that could be construed as a potential conflict of interest.
